# Cost of illness of the stomach cancer in Japan - a time trend and future projections

**DOI:** 10.1186/1472-6963-13-283

**Published:** 2013-07-23

**Authors:** Kayoko Haga, Kunichika Matsumoto, Takefumi Kitazawa, Kanako Seto, Shigeru Fujita, Tomonori Hasegawa

**Affiliations:** 1 Department of Social Medicine, Toho University School of Medicine, 5-21-16 Omori-nishi, Ota-ku, Tokyo 143-8540, Japan

**Keywords:** Cost of illness (COI), Stomach cancer, Health economics, Health policy, Aging

## Abstract

**Background:**

Stomach cancer is one of the leading causes of cancer deaths in Japan. The objectives of this study were to estimate and project the economic burden associated with stomach cancer in Japan, and to identify the key factors that drive the economic burden of stomach cancer.

**Methods:**

We calculated Cost of illness (COI) of 1996, 2002, 2008, 2014 and 2020 by using government office statistics and the COI method. We calculated direct cost and indirect cost (morbidity cost and mortality cost), and estimated the COI by summing them up.

**Results:**

The number of deaths remained at approximately 50,000 in 1996–2008. COI was in downward trend from 1,293.5 billion yen in 1996 to 1,114.2 billion yen in 2008. Morbidity cost was 85.6 billion yen and 54.0 billion yen, mortality cost was 972.3 billion yen and 806.4 billion yen, and mortality cost per person was 19.4 million yen and 16.1 million yen in 1996 and 2008, respectively. Decrease of mortality cost that accounted for a large part of the COI (72.4% in 2008) was the major contributing factor. COI is predicted to decrease if the trend of health related indicators continues (442.8-1,056.1 billion yen depending on the model in 2020). Mortality cost per person is also predicted to decrease (9.5-12.5 million yen depending on the model in 2020).

**Conclusions:**

If the trend of health related indicators continues, it is estimated that COI of stomach cancer would decrease. “Aging”, “change of the healthcare providing system” and “new medical technology” are considered as contributing factors of COI.

## Background

Stomach cancer (ICD 10 cord: C16) was the leading cause of cancer deaths in Japan, but recently its mortality rate has been decreasing. The age-adjusted mortality rate has been continuously decreasing from 56.9 in 1975 to 18.1 in 2010[1]. The age-adjusted morbidity rate has also been continuously decreasing. It decreased from 84.0 in 1975 to 51.3 in 2007 [1]. However, the number of deaths during these two periods has remained the same and approximately 50,000 people die of stomach cancer every year. In 2009 stomach cancer accounted for 14.5% of all cancer deaths. Stomach cancer was the 2^nd^ leading cause of cancer deaths next to lung cancer in men and the 3^rd^ leading cause of deaths next to colon cancer and lung cancer in women in Japan. Even today, mortality rate of stomach cancer remains high. Furthermore, according to “Survey of National Medical Care Insurance Services”, the medical expenses due to the stomach cancer treatment accounted for approximately 10% of the medical expenses of all cancers in 2009.

Stomach cancer is a disease of high morbidity rate in the elderly, and with Japan’s acceleration into an aging society, the economic burden related to stomach cancer is considered to be changing dramatically. However, only a few articles estimate its economic burden in Japan. Most of them, moreover, are limited to the estimation of direct medical expenses, only in one time-point or cost-effectiveness of *Helicobacter pylori* eradication [2-4]. Koinuma estimated that cost (direct cost, morbidity cost and mortality cost) of stomach cancer was approximately 1,400 billion yen in 2005 [3]. The incidences of stomach cancer differ greatly by country. It is known to be high in Japan and to be low in the United States and Europe. Low incidence and limited social burden might explain for the scarcity of studies about cost of stomach cancer in those countries. Furthermore there are few long-term estimations or future predictions of economic burden of stomach cancer.

The objectives of this study were to estimate and project the economic burden associated with stomach cancer in Japan, and to identify the key factors that drive the economic burden of stomach cancer.

## Methods

### Analysis method

In this study, we used government office statistics and the Cost of illness (COI) method developed by Rice DP to estimate burden of disease [5-10].

The COI consists of direct cost (DC) and indirect cost (IC). Indirect cost consists of morbidity cost (MbC) and mortality cost (MtC). COI is calculated using following equations:

COI=DC+MbC+MtC

•Direct cost

•The direct cost is defined as medical expenses (treatment costs, hospital charges, laboratory costs, drug costs, etc.). In this study, we used “Survey of National Medical Care Insurance Services”, and calculated annual medical expenses based on reimbursement data.

•Indirect cost

•The indirect cost is an opportunity cost lost by contraction of a disease and the death. They are calculated using following equations:

MbC=TOVy×LVd/2+THD×LVd

MtC=NDy×LVl

•Where: TOVy is total person-days of outpatient visit, LVd is one day labor-value per person, THD is total person-days of hospitalization, NDy is the number of deaths, and LVl is lifetime labor-value per person.

•Total person-days of outpatient visit and hospitalization according to sex and 5 years age groups were calculated based on “Patient Survey”. The labor-value was calculated according to sex and 5 years age groups by using “Basic Survey on Wage Structure”, “Labor Force Survey” and “Estimates of monetary valuation of unpaid work” [11]. Lifetime labor-value was calculated by summing up the income which they could have earned in the future if they had not died. We calculated the morbidity cost by assuming that one day labor-value loss for one hospitalized day and a half day labor-value loss for one outpatient visit. We used the number of deaths by stomach cancer according to sex and 5 years age groups from “Vital Statistics”. LVd and THD were obtained as follows:

$$\mathrm{LVd}=\left(\mathrm{Iy}+\mathrm{ULVy}\right)/365$$

THD=HPy×ALOS

•Where: Iy is annual income per person, ULVy is annual monetary valuation of unpaid work per person, HPy is the number of annual hospitalized patients, and ALOS is average length of stay.

•We assumed an average life expectancy for their life span. Future labor-value was adjusted to a present value using 3% discount rate, because 3% is widely used as discount rate in the United States, where the application of the COI method was popular.

### Examination element

The following 8 elements were considered to affect COI; (1) number of times of outpatient visits, (2) number of times of hospitalization, (3) average length of stay, (4) number of deaths, (5) human capital value, (6) medical technology, (7) income, (8) discount rate used to calculate lifetime income. They seem to interact with one another. In this study, we tried to make clear the factors relating to COI by examining these elements. As for (6), we used only fatality rate calculated from morbidity rate and mortality rate as an indicator of medical technology only for discussion since it was not used for calculation of COI, and the change of specific treatment regimen was not taken into consideration.

### Time series estimation of COI

At first, using available past data, we estimated COI in 1996, 2002 and 2008. Then, we predicted COI in 2014 and 2020 using two methods.

The first method was “fixed model estimation”, that is, the estimation that assumed health related indicators (mortality rate, number of times of outpatient visit per population, number of times of hospitalization per population, and average length of stay) were fixed. We used those values at 2008, and only future population estimation was used as a variable. First, we calculated mortality rate, number of times of outpatient visit per population, and number of times of hospitalization per population according to sex and 5 years age groups at 2008 as indicators of the standard year. Next, by multiplying them with the future population estimates according to sex and 5 years age groups of 2014 and 2020, we calculated the predictive number of deaths, total person-days of outpatient visit, and total person-days of hospitalization in 2014 and 2020. We estimated the morbidity cost and mortality cost of 2014 and 2020 by using the data of average length of stay, life expectancy and labor-value at 2008. Direct costs were calculated by multiplying the cost of outpatient visit and hospitalization expenses at 2008 with the rate of change of the total number of days of outpatient visit and hospitalization of 2014 and 2020.

The second method was “variable model estimation”, that is, the estimation where health related indicators changed at the same pace as in the past 12 years, in addition to the change of population and age structure. First, we drew the trend line (a logarithm approximation and a linear approximation) since 1996 of each indicator, respectively. Next we calculated the value of the 2014 and 2020 using the trend line formula. We used three variable models according to the approximation: 1. Logarithm model; health related indicators were calculated using a logarithm approximation. 2. Linear model; health related indicators were calculated using a liner approximation. 3. Mixed model; an approximation with the higher coefficient of determination. The elements used for calculation in future prediction of COI are shown in Table 1.

**Table 1:** The elements used for calculation in future prediction of Cost of illness (COI)

**Model**	** Item**	**Elements used for calculation**	**Fixed or Varied**
Fixed model	Number of deaths	Mortality rate	Fixed
		The population estimates	Varied
	Direct cost	The expenses of outpatient visit and hospitalization	Fixed (Calculated using the unit cost in 2008)
		Total person-days of outpatient visit	Varied
		Total person-days of hospitalization	Varied
	Morbidity cost	Number of times of outpatient visit per population	Fixed
		Number of times of hospitalization per population	Fixed
		Average length of stay	Fixed
		The population estimates	varied
		Labor-value	Fixed (one day labor-value loss for one hospitalized day and a half day labor-value loss for one outpatient visit)
	Mortality cost	Number of deaths	Varied
		Life expectancy	Fixed
		Labor-value	Fixed
		Discount rate: 3%	Fixed
Variable model •Logarithm model •Linear model •Mixed model	Number of deaths	Mortality rate	Varied (Calculated using the trend line formula)
		The population estimates	Varied
	Direct cost	The expenses of outpatient visit and hospitalization	Fixed (Calculated using the unit cost in 2008)
		Total person-days of outpatient visit	Varied
		Total person-days of hospitalization	Varied
	Morbidity cost	Number of times of outpatient visit per population	Varied (Calculated using the trend line formula)(minimum value: the previous value before 0)
		Number of times of hospitalization per population	Varied (Calculated using the trend line formula)(minimum value: the previous value before 0)
		Average length of stay	Varied (Calculated using the trend line formula)(minimum value: 8.2days)
		The population estimates	Varied
		Labor-value	Fixed (one day labor-value loss for one hospitalized day and a half day labor-value loss for one outpatient visit)
	Mortality cost	Number of deaths	Varied
		Life expectancy	Fixed
		Labor-value	Fixed
		Discount rate: 3%	Fixed

Fixed: The value of 2008 was used. Varied: The values of 2014 and 2020 were calculated based on the trend line.

2008 data was used for life expectancy and the labor-value. In addition, we used 1996, 2002 and 2008 “Population estimates” by Ministry of Internal Affairs and Communications, and 2014 and 2020 “Population statistics of Japan” by National Institute of Population and Social Security Research.

When estimating using the trend line, a future predicted value sometimes took less than 0. Therefore, we needed to set a “minimum value”. For mortality rate, number of times of outpatient visit per population and number of times of hospitalization per population, the previous value before 0 was set as “minimum value” where there were minus values. As for average length of stay, we set 8.2 days- the average length of stay of patients with malignant neoplasm (2006) of OECD 28 countries- as “minimum value”. In Japan, the average length of stay of stomach cancer (26.8 days) was similar to that of the whole malignant neoplasm (23.9 days) according to “Patient Survey”.

## Results

### Result of the estimation of time series COI in 1996, 2002 and 2008

COI was estimated at 1,293.5 billion yen in 1996, 1,234.4 billion yen in 2002 and 1,114.2 billion yen in 2008. Comparing 1996 and 2008, COI decreased by 13.9%. According to “Patient Survey”, the number of total patients with stomach cancer has been decreasing; 305,000 people in 1996, 222,000 in 2002 and 213,000 in 2008. And the average length of stay has been shortening; 47.1 days, 39.3 days and 26.8 days, respectively. The number of deaths has not changed so much, but the average age of deaths has been gradually rising, and the proportion of deaths of 65 years or older among the whole deaths has increased (Table 2). Fatality rate of both sexes has decreased possibly reflecting the progress of the recent medical technologies. However, fatality rate of elderly people was higher than that of young people, and decreased less compared to that of young people (fatality rate of people aged 65 or older was 0.63 in 1996 and 0.58 in 2002, and that of people aged 64 or younger was 0.40 in 1996 and 0.34 in 2002). The direct cost has increased a little, but morbidity cost and mortality cost have decreased. The mortality cost per person (mortality cost divided by number of deaths) has decreased. In mortality cost, the proportion of 65 years or older has increased.

**Table 2:** The time trend of Cost of illness (COI) of stomach cancer

**Item**		**1996**	**2002**	**2008**
Population (thousand person)		125,865	127,433	127,690
[% of 65 years or older]		15.1%	18.5%	22.1%
Number of stomach cancer deaths (person)		50,161	49,211	50,156
[% of 65 years or older]		70.1%	76.2%	80.7%
Average age of death (year)		70.2	72.2	74.4
Crude morbidity rate (per 100 thousand)	male	109.3	115.1	NA
	female	52.5	53.9	NA
Crude mortality rate (per 100 thousand)	male	53.0	51.6	53.7
	female	28.0	27.1	26.6
Fatality rate	male	0.48	0.45	NA
	female	0.53	0.50	NA
Direct cost (billion yen)		235.5	251.9	253.7
Morbidity cost (billion yen)		85.6	63.8	54.0
Mortality cost (billion yen)		972.3	918.7	806.4
[% of 65 years or older]		24.9%	37.7%	42.0%
Mortality cost per person (million yen)		19.4	18.7	16.1
COI (billion yen)		1,293.5	1,234.4	1,114.2

Source of population:Ministry of Internal Affairs and Communications “Population Estimates”.

Source of the number of stomach cancer deaths: “Vital Statistics”.

Average age of death: Calculated according to the number of deaths, sex and age (5 years old age-grade), cause of death in “Vital Statistics”.

Source of crude morbidity rate and crude mortality rate:Center for Cancer Control and Information Services, National Cancer Center, Japan.

Fatality rate:We calculated by dividing the crude mortality rate by crude morbidity rate.

NA: not available

### Result of the estimation of COI in 2014 and 2020 (fixed model)

Future projection of Japanese population (medium estimate) by National Institute of Population and Social Security Research predicted a gradual decrease (127.6 million in 2008, 125.9 million in 2014 and 122.7 million in 2020), and a proportion of 65 years old or older was predicted to gradually increase (22.1%, 26.2% and 29.2%, respectively) [12](Table 3). The number of deaths of stomach cancer was expected to increase by 31.9% from 2008 to 2020. Specifically the number of deaths of elderly people (65 years or older) increases remarkably, and it occupies 87.2% of all stomach cancer deaths in 2020. The average death age was expected to rise. The mortality cost per person showed downward tendency. The proportion of 65 years or older would occupy 50.0% of all mortality cost in 2020. In the fixed model, direct cost, morbidity cost and mortality cost would increase, and we estimated that COI would increase by 9.8% from 2008 to 2020 (1,177.4 billion yen in 2014 and 1,224.3 billion yen in 2020).

**Table 3:** Future prediction of Cost of illness (COI)

**Model**		**Item**	**2008**	**2014**	**2020**
		Estimated population (thousand person)	127,690	125,862	122,735
		[% of 65 years or older]	22.1%	26.2%	29.2%
Fixed model		Number of stomach cancer deaths (person)	50,156	58,784	66,164
		[% of 65 years or older]	80.7%	84.7%	87.2%
		Average age of death (year)	74.1	75.5	76.6
		Direct cost (billion yen)	253.7	288.0	315.3
		Morbidity cost (billion yen)	54.0	56.1	57.3
		Mortality cost (billion yen)	806.4	833.4	851.7
		[% of 65 years or older]	42.0%	47.3%	50.0%
		Mortality cost per person (million yen)	16.1	14.2	12.9
		COI (billion yen)	1,114.2	1,177.4	1,224.3
Variable model	Logarithm model	Number of stomach cancer deaths (person)	50,156	58,554	62,868
		[% of 65 years or older]	80.7%	84.9%	87.7%
		Average age of death (year)	74.1	75.6	76.9
		Direct cost (billion yen)	253.7	243.8	227.9
		Morbidity cost (billion yen)	54.0	49.3	42.4
		Mortality cost (billion yen)	806.4	824.4	785.8
		[% of 65 years or older]	42.0%	47.5%	51.0%
		Mortality cost per person (million yen)	16.1	14.1	12.5
		COI (billion yen)	1,114.2	1,117.5	1,056.1
	Linear model	Number of stomach cancer deaths (person)	50,156	45,021	37,581
		[% of 65 years or older]	80.7%	87.1%	91.8%
		Average age of death (year)	74.1	76.7	79.1
		Direct cost (billion yen)	253.7	162.3	67.3
		Morbidity cost (billion yen)	54.0	31.0	17.7
		Mortality cost (billion yen)	806.4	557.0	357.7
		[% of 65 years or older]	42.0%	52.5%	60.7%
		Mortality cost per person (million yen)	16.1	12.4	9.5
		COI (billion yen)	1,114.2	750.3	442.8
	Mixed model	Number of stomach cancer deaths (person)	50,156	45,021	37,581
		[% of 65 years or older]	80.7%	87.1%	91.8%
		Average age of death (year)	74.1	76.7	79.1
		Direct cost (billion yen)	253.7	187.1	100.4
		Morbidity cost (billion yen)	54.0	37.1	26.4
		Mortality cost (billion yen)	806.4	557.0	357.7
		[% of 65 years or older]	42.0%	52.5%	60.7%
		Mortality cost per person (million yen)	16.1	12.4	9.5
		COI (billion yen)	1,114.2	781.2	484.5

Source of estimated population:2008;Ministry of Internal Affairs and Communications “Population Estimates” 2014•2020;National Institute of Population and Social Security Research “Population Statistics of Japan”.

### Result of the estimation of COI in 2014 and 2020 (variable model)

**Figure 1:**
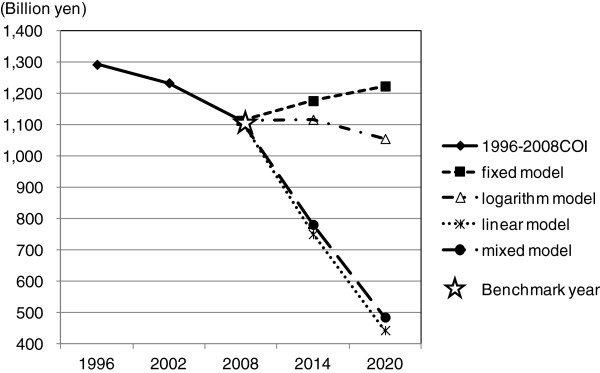
The time trends of Cost of illness (COI) by prediction models.

**Figure 2:**
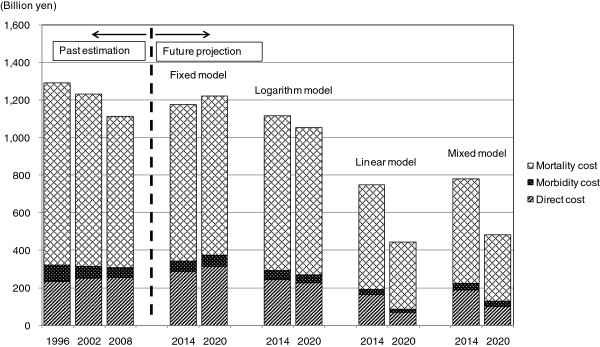
Cost of illness (COI) projection with cost element.

Figures 1 and 2 show the trends of COI of each model. Any model but the fixed model showed a downward tendency.

Future prediction of COI is shown in Table 3. In the logarithm model, the number of predictive deaths increased, however, the number was less than that of the fixed model. Direct cost and morbidity cost decreased continuously, and mortality cost increased in 2014, but decreased in 2020. As a result, COI also increased to 1,117.5 billion yen in 2014, but decreased to 1,056.1 billion yen in 2020.

In the linear model, the number of predictive deaths was estimated to show a decrease. Direct cost, morbidity cost, mortality cost and COI were also estimated to decrease continuously from 2008. COI was estimated to 750.3 billion yen in 2014 and 442.8 billion yen in 2020.

Since the trend of each health related indicator was different, the monotype estimation (both logarithmic model and linear model) might not predict future COI precisely. Therefore we developed a mixed model where we adopted value with higher coefficient of determination in each age group. In the mixed model, the logarithm approximation was used for number of times of outpatient visit and number of times of hospitalization per population, and the linear approximation was used for average length of stay and mortality. This adaptation was made to equal the number of deaths and mortality cost to those of the linear model. In the mixed model, COI was estimated to decrease to 781.2 billion yen in 2014 and 484.5 billion yen in 2020, and it decreased by 56.5% from 2008 to 2020. Number of deaths, direct cost, morbidity cost, mortality cost and COI showed downward trend from 2008, and were estimated to be less than those of the logarithm model, but were estimated to exceed those of the linear model. In 2020 the proportion of deaths of 65 years or older amounted to 91.8% of all deaths, and average age of death rose to 79.1 years old. Also the mortality cost per person showed remarkable decline (9.5 million yen), and the proportion of mortality cost of 65 years or older would account for 60.7%.

## Discussion

We found that COI was in downward trend from 1996 to 2008. As for future projection, only the fixed model suggested a slight increase of COI. With the variable models where health related indicators’ change was taken into consideration, COI was expected to decrease. Specifically, the mixed model suggested continuous decrease of the number of deaths, direct cost, morbidity cost, mortality cost and COI. Since the mixed model was a combination of models of higher coefficient of determination, it was considered that the mixed model was the most valid model for our study.

In our analysis, the main model is the mixed model. The fixed model is a reference. The logarithm model is the low-end, and the linear model is the high-end estimation, respectively, and they can be regarded as sensitivity analyses showing the robustness of the mixed model.

Aging population, change in providing system, and change in medical technology could affect COI. We observed (1) decrease of number of times of outpatient visit per population, (2) decrease of number of times of hospitalization per population, and (3) shortening of average length of stay, which could all have contributed to the decrease of COI.

Japanese society has been aging rapidly in recent years. Among elderly people, morbidity and mortality of stomach cancer were high, and health related factors were also related to aging. For example, fatality rate of the elderly people was high and had decreased less compared to that of young people, and “aging” contributed to increase the number of deaths of stomach cancer in total. On the other hand, labor- value of elderly people was low, and human capital value per person was low, thus contributing to decreased mortality cost. Recent COI decrease can be explained by the balance between “decrease of human capital loss by decreased number of deaths in young people” and “increase of the human capital loss by increased number of deaths in elderly people”.

Furthermore, “aging” is predicted to accelerate in the future [12]. This will affect future COI. The fixed model showed that all of the number of predictive deaths, direct cost, morbidity cost, mortality cost and COI would increase in the future. Even though the number of deaths was estimated to increase by 31.9% from 2008 to 2020, mortality cost was estimated to increase only by 5.6%. Elderly people aged 65 years or older would occupy a significant portion of deaths (80.7% in 2008, 87.2% in 2020). Mortality cost per person would decrease since the human capital value would decrease according to age of deaths (16.1 million yen in 2008, 12.9 million yen in 2020). Although mortality cost would increase by the rapid increase of the number of deaths especially in elderly people, the increase rate of the total mortality cost would remain low.

In the mixed model, it was estimated that all of the number of predictive deaths, direct cost, morbidity cost, mortality cost and COI would decrease in the future. The number of deaths would decrease by 25.1%, and mortality cost would decrease by 55.6% from 2008 to 2020. Compared with the fixed model and the logarithm model, increase of the proportion of the elderly people among all deaths (80.7% in 2008, 91.8% in 2020), rise of the average death age, decrease of mortality cost per person (16.1 million yen in 2008, 9.5 million yen in 2020) predicted in this model were remarkably different. The fact that the number of deaths in young people whose human capital value was high decreased significantly may be considered to have caused the decrease of the total mortality cost.

Recently, the Japan Ministry of Health, Labor and Welfare introduced several policies as a part of a health sector reform [13]. Most policies aimed at cost-efficient care by containing hospital use. Among them, the introduction of DPC/PDPS (Diagnosis Procedure Combination/Per Diem Payment System) in 2003 encouraged hospitals to shorten length of hospital stay. With DPC/PDPS, hospitals with longer length of hospital stay were paid less money, and hospitals reimbursed by DPC/PDPS were likely to introduce clinical pathways, increase the use of outpatient department for diagnostic tests, procedures and chemotherapies, and further establish network with other step-down facilities to shorten length of stay [14]. New medical technologies have made outpatient treatment of stomach cancer possible. More and more stomach cancer patients are being treated as outpatients and in their homes. In this study, a slight increase was observed in number of times of outpatient visit per population from 2005 to 2008. Although specific medical technologies were out of the scope of this study, containment of hospital use and development of new technologies could contribute to the little change or the increased use of outpatient services.

The results of this study suggested that the decrease of the human capital value by aging was the main factor of the change of COI in stomach cancer and other factors such as healthcare providing system and new medical technologies could also influence COI. Although aging of the society cannot be controlled in the near future, the latter two can be manipulated by a change in health policy and progress of medical technology. Therefore, the detailed and continuous analyses of these are important.

This study still has its limitations. The COI method was used in our study since it has been used widely to evaluate the burden of disease since 1960s, and has been used for policymaking [15-19]. There are reports that the COI method has been used in several countries and international organization [20,21]. On the other hand, there is also the criticism against the COI method that COI studies can be used for advocacy purposes and it cannot be used for policy making or decision-making [22]. We consider that the COI method is suitable for time series comparison and future prediction of the economic burden because of its simplicity, admitting the availability and effectiveness in policy-making are still to be demonstrated. Among the other limitations is how a value of estimations using approximate curves reflects real situation in predicting the future. We should be careful in interpreting the outcome since the data used were collected from a relatively short term during the period between 1996 and 2008. The appropriateness of setting the “minimum value” should also be examined. Long term insurance was introduced in 2000, and shift of healthcare expenditure from medical insurance to long term insurance occurred. This study dealt with COI of stomach cancer, and assuming most patients were being treated by medical insurance, cost of long term insurance was not taken into account. In some chronic diseases, cost of long term insurance should be included in COI. Furthermore, in the COI method, it is difficult to consider the quality of life of the patients, and it is also difficult to include a change of utility and efficiency of a specific treatment into analysis.

The COI method enables to clarify how to use the limited health resources effectively and supports rational decision making by measuring a burden of disease by monetary term [6]. It can justify intervention plan and provide basic information for policy making in the prevention and management of the disease [10].

Cancer has become the leading cause of death in Japan since 1981. It is not just a serious problem in the life and health of a people, but its economic burden should also be considered. The government took action by implementing various cancer control programs beginning with “Ten-year General Anti-cancer strategy” (1984). The degree of contribution or the resulting effects on the population by each governmental cancer control program could not be assessed by this study, but mortality and COI of stomach cancer has been in the tendency to decrease. In recent years, as the measure for stomach cancer control, *Helicobacter pylori* eradication and new screening method were introduced [23,24]. There are several studies reporting economic effects of *Helicobacter pylori* eradication [4,25], and *Helicobacter pylori* eradication might reduce healthcare cost of stomach cancer. Our study does not take the effect of a specific treatment technology into consideration, and further study of COI including the possible effect of *Helicobacter pylori* eradication treatment would be needed.

## Conclusions

We confirmed that the future prediction of COI of stomach cancer was possible by using government office statistics. COI was in a downward trend from 1996 to 2008. If the trend of health related indicators continue, it is estimated that COI of stomach cancer would decrease. “Aging”, “change of the healthcare providing system” and “new medical technology” are considered as contributing factors of COI, and in particular, it was revealed that the decrease of the human capital value by aging has salient influence on the COI. It is anticipated that future studies of COI of stomach cancer to include the targeted effects of new cancer control programs to enable prioritizing implementation of new policies and the development of medical technologies.

## Abbreviations

COI: Cost of illness; DC: Direct cost; IC: Indirect cost; MbC: Morbidity cost; MtC: Mortality cost; LVd: One day labor-value per person; LVl: Lifetime labor-value per person; TOVy: Total person-days of outpatient visit; THD: Total person-days of hospitalization; NDy: Number of deaths; Iy: Annual income per person; ULVy: Annual monetary valuation of unpaid work per person; HPy: Number of annual hospitalized patients; ALOS: Average length of stay.

## Competing interests

The authors declare that they have no competing interests.

## Authors’ contributions

KH participated in the design of the study, performed the data collection and the analysis, and drafted the manuscript. KM participated in the design of the study and performed the analysis. TK, KS and SF performed the data collection and the analysis. TH conceived of the study, and participated in its design and helped to draft the manuscript. All authors read and approved the final manuscript.

## Pre-publication history

The pre-publication history for this paper can be accessed here:

http://www.biomedcentral.com/1472-6963/13/283/prepub
